# Molecular basis of chemosensitivity of platinum pre-treated ovarian cancer to chemotherapy

**DOI:** 10.1038/sj.bjc.6605817

**Published:** 2010-08-10

**Authors:** S Glaysher, F G Gabriel, P Johnson, M Polak, L A Knight, K Parker, M Poole, A Narayanan, I A Cree

**Affiliations:** 1 Translational Oncology Research Centre, Queen Alexandra Hospital, Portsmouth PO6 3LY, UK; 2 School of Computing, University of Portsmouth, Buckingham Building, Portsmouth PO1 3HE, UK; 3 School of Computing and Mathematical Sciences, Auckland University of Technology, Private Bag 92006, Auckland 1142, New Zealand

**Keywords:** ovarian cancer, chemosensitivity, chemoresistance, gene expression, ATP, RT–PCR

## Abstract

**Background:**

Ovarian cancer shows considerable heterogeneity in its sensitivity to chemotherapy both clinically and *in vitro*. This study tested the hypothesis that the molecular basis of this difference lies within the known resistance mechanisms inherent to these patients' tumours.

**Methods:**

The chemosensitivity of a series of 31 ovarian tumours, all previously treated with platinum-based chemotherapy, was assessed using the ATP-based tumour chemosensitivity assay (ATP-TCA) and correlated with resistance gene expression measured by quantitative reverse-transcriptase polymerase chain reaction (qRT-PCR) in a TaqMan Array following extraction of mRNA from formalin-fixed paraffin-embedded tissue. The results were standardised against a housekeeping gene (*PBGD*), and assessed by multiple linear regression.

**Results:**

Predictive multiple linear regression models were derived for four single agents (cisplatin, gemcitabine, topotecan, and treosulfan), and for the combinations of cisplatin+gemcitabine and treosulfan+gemcitabine. Particularly strong correlations were obtained for cisplatin, gemcitabine, topotecan, and treosulfan+gemcitabine. No individual gene expression showed direct correlation with activity in the ATP-TCA. Genes involved in DNA repair and apoptosis were strongly represented, with some drug pumps also involved.

**Conclusion:**

The chemosensitivity of ovarian cancer to drugs is related to the expression of genes involved in sensitivity and resistance mechanisms.

The variable response to chemotherapy in ovarian cancer is well recognised clinically. Patients crossing from one regimen to the alternative in clinical trials often show responses (Piccart *et al*, 2000; Muggia, 2003). This suggests that it might be possible to optimise therapy if it was also possible to develop a test to determine which regimen would be most effective for an individual patient. We and others have used cellular chemosensitivity tests to show similar heterogeneity in ovarian cancer, and clinical comparisons suggest that these tests correlate relatively well with outcome (Andreotti *et al*, 1995; Kurbacher *et al*, 1998; Konecny *et al*, 2000; Sharma *et al*, 2003; Cree *et al*, 2007) despite the rapid development of resistance in many patients (Di Nicolantonio *et al*, 2005). Clinical use of such tests is more problematic and requires access to specialist laboratory facilities, as well as a large amount of fresh tumour tissue. These assays have therefore failed to be widely accepted into clinical practice.

Molecular assays offer the prospect of developing predictive assays capable of wider use. However, studies of single genes have shown little predictive efficacy, unless they happen to be the targets of the drugs concerned. Multi-gene signatures are likely to be required. There are two feasible approaches to the generation of multi-gene signatures for predictive testing. The first is to screen very large numbers of genes using hybridisation arrays, correlate these with clinical outcome, and apply sophisticated statistical methods to generate predictive gene signatures (Wigle *et al*, 2002; Potti *et al*, 2006). The second is to take an hypothesis-driven approach to generate gene sets based on knowledge of the pathways involved in resistance and sensitivity to individual drugs that can then be correlated with *in vitro* chemosensitivity data or clinical outcome (Kikuchi *et al*, 2003). We have taken the latter approach and have designed gene sets for chemosensitivity prediction based on published reports and previous studies.

In ovarian cancer, platinum (usually carboplatin) is the mainstay of treatment in the primary setting, with single-agent response rates around 60% (Lokich and Anderson, 1998). Single agents such as paclitaxel, topotecan, and liposomal doxorubicin are used as second-line treatments with response rates around 20% (Sharma *et al*, 2003). Other drugs such as alkylating agents (e.g. treosulfan) also have activity and we have shown that the combination of treosulfan+gemcitabine is particularly active (Cree, 2003; Sharma *et al*, 2003). Combination of gemcitabine with platinum, even following previous treatment is effective due to the ability of gemcitabine to reverse resistance associated with enhanced DNA repair (Cree, 2003).

The mechanisms of resistance to these agents have been studied in some detail. Clinical studies using genome-wide gene expression arrays have been performed and have shown signatures associated with resistance to a number of agents (Richardson and Kaye, 2005; Tsuda *et al*, 2005; Olivier *et al*, 2006). It is generally assumed that the same resistance mechanisms used by cells to circumvent single-agent activity will also be used against combinations of these drugs. However, there are few mechanistic studies of resistance to the combinations used in ovarian cancer to confirm or refute this supposition.

This study has tested the hypothesis that the molecular basis of the observed heterogeneity of chemosensitivity of ovarian cancer is determined by the known resistance mechanisms expressed by these patients' tumours. Resistance to anti-cancer drugs involves many mechanisms (Cree *et al*, 2002a, 2002b), and an array-based approach to study the genes involved is therefore sensible. The mechanisms of cellular (i.e. non-pharmacokinetic) resistance to chemotherapy include down-regulation of target expression, drug metabolism, membrane-located xenobiotic pumps, altered susceptibility to apoptosis, and altered growth/cell cycle or differentiation. Knowledge of these pathways enabled us to design a TaqMan Array microfluidic quantitative reverse-transcriptase PCR (qRT-PCR) card to include 91 genes known to be involved in drug resistance/sensitivity to anthracyclines, 5-FU, alkylating agents, and taxanes. The card also included five housekeeping genes to allow standardisation of the results for comparison of individual tumour data. The gene set chosen was not intended to be comprehensive, but to establish proof of principle for the use of *in vitro* sensitivity data with gene expression data to determine the contribution of individual genes to drug sensitivity and resistance in ovarian cancer.

## Patients and methods

In this study we have used qRT-PCR to examine the expression of genes previously shown to be involved in resistance to chemotherapy with platinum, anthracyclines, taxanes, and alkylating agents in ovarian cancer. The expression profiles have been compared with quantitative chemosensitivity data obtained for the same tumours using the ATP-based chemosensitivity assay tumour chemosensitivity assay (ATP-TCA).

### Patients and samples

A series of 31 ovarian tumour samples were obtained from surgical specimens of platinum-pre-treated patients debulking surgery or at staging laparoscopy with written consent from 2001 to 2006. Ethics committee approval was obtained and the study conformed to the Declaration of Helsinki Principles. The fresh samples were used to obtain sensitivity data, and patients also had formalin-fixed paraffin-embedded (FFPE) material taken for histology. This provided a source of material for qRT-PCR. The age of patients ranged from 18 to 57 years (average 38 years), and all were FIGO stage 3c or IV. Histologically, 10 tumours were classified as papillary serous, 4 as clear cell, 3 endometrioid, 1 mixed mullerian, 1 mucinous, and the remaining 12 as poorly differentiated by the reporting pathologist. In 12 cases paclitaxel was given in addition to platinum as first-line treatment, 1 case received cisplatin+paclitaxel, and the remainder received carboplatin alone. Twelve cases presented with early recurrence during or within 6 months of initial treatment consistent with clinical resistance to platinum. Nine patients were relapse cases following second-line treatment, whereas the remainder were first relapse cases.

### ATP-TCA

The ATP-TCA was performed as previously reported (Andreotti *et al*, 1995; Cree, 1998). Samples from solid tumours were transported to the laboratory in 25 ml specimen bottles containing cooled transport medium consisting of DMEM (Sigma, Poole, Dorset, UK) with added antibiotics. Tumour cells were obtained by enzymatic dissociation, washed in a serum-free complete assay medium (CAM; available from DCS, Hamburg, Germany), and purified by density centrifugation to remove debris. The cells were washed, re-suspended in CAM, and plated in 96-well polypropylene plates at 20 000 cells per well with six doubling dilutions of four drugs or combinations, tested in triplicate from a 200% test drug concentration (TDC) to 6.25% TDC. The concentrations of the drugs used are given in Table 1. One row of each plate contained medium only and one row a maximum inhibitor control. The plates were incubated for 6 days at 37°C with 5% CO_2_, following which ATP was extracted with tumour cell extraction reagent (DCS). Aliquots of the extract were transferred to a white 96-well polystyrene plate to which an equal amount of luciferin-luciferase was added. The resulting luminescence was read in a luminometer (MPLX; Berthold Diagnostic Systems GmbH, Pforzheim, Germany) and the data were transferred to an Excel (Microsoft) spreadsheet for analysis. The results were expressed as the percentage inhibition at each concentration tested compared with an untreated control, and a summary index was calculated as the sum of the surviving cell fraction at each concentration calculated as 600−Sum(Inhibition_600_:Inhibition_6.25_) for comparison with qRT-PCR results, where Index_SUM_=0 indicates complete inhibition and Index_SUM_=600 indicates no effect (Hunter *et al*, 1993).

### Extraction of mRNA from FFPE tumor tissue

The H&E-stained sections of selected FFPE blocks of ovarian tumor tissue, previously marked with tumour areas of interest (AOI) by a pathologist, were matched and overlaid onto the corresponding FFPE block to give >80% neoplastic cells, comparable with the ATP-TCA (Andreotti *et al*, 1995). The manual tissue arrayer (MTA1; Beecher Instruments Inc., Sun Prairie, WI, USA) fitted with a punch stylet 1.0 mm in diameter was aligned over the desired AOI, which was punched out from the block. The stylet was decontaminated (Ambion DNA and RNA Zap) and cleaned (70% alcohol) between each FFPE block. A minimum of two 1.0 mm punches were obtained from each block and placed in a sterile labelled 1.5 ml microcentrifuge tube.

The remainder of the extraction process was performed as previously reported (Glaysher *et al*, 2009). In brief, the tubes were heated at 70°C in a Stuart SBH200D heating block (Bibby Scientific Limited, Stone, Staffordshire, UK) for 20 min. Excess paraffin wax was subsequently removed and xylene was added to the tube for 10 min at 50°C. The microfuge tube was removed from the heating block, centrifuged at 12 000 **g** for 2 min in a Sanyo MSE Micro Centaur centrifuge (MSE (UK) Ltd, Lower Sydenham, London, UK) and waste xylene was removed. The samples were washed two additional times with xylene. Residual xylene was removed with 100% ethanol and the tubes were allowed to stand for 10 min at room temperature. Following centrifugation at 12 000 **g** for 5 min, the ethanol was removed by pipette, and the process was repeated once more. The microfuge tube lids were opened to allow evaporation of any residual ethanol at 50°C for 10 min, before protease digestion.

Protease digestion and RNA extraction were performed using an Ambion RecoverAll kit (catalogue no. AM1975; Applied Biosystems, Life Technologies, Carlsbad, CA, USA.) according to the manufacturer's instructions as previously described (Glaysher *et al*, 2009). The lysates resulting from protease digestion were stored at −20°C before RNA extraction.

### Two-step RT–PCR

Reverse transcription (RT) was performed using an High-Capacity cDNA Archive Kit (catalogue no. 4322171; Applied Biosystems) according to the manufacturer's instructions and as previously reported (Glaysher *et al*, 2009) in a 0.2 ml PCR tube. The final RNA concentration in the RT mix was 1–20 ng *μ*l^−1^. Cycling conditions were step 1, 25°C for 10 min, step 2, 37°C for 120 min. After removal from the thermal cycler, the tubes were pulse spun in a microfuge at 12 000 **g** for 30 s and stored overnight at 4°C. The following day cDNA content was measured using a NanoDrop spectrophotometer before use in the TaqMan Array.

TaqMan Arrays (Applied Biosystems) were run according to the manufacturer's instructions (Glaysher *et al*, 2009). Samples were made up with TaqMan × 2 Universal Master Mix with UNG Amperase (catalogue no. 4364338; Applied Biosystems) and mixed with an equal volume of cDNA to give a final concentration of 300 ng *μ*l^−1^ DNA. All four samples were then each pipetted into two ports (100 *μ*l per port) of the 384-well card, for the 96 genes arrayed in a Chemosensitivity Gene Expression Array (CGEA-1; CanTech Ltd., Portsmouth, UK). The genes on each card are given in Table 2. The loaded card was then placed, port upwards, into a balanced centrifuge (type, address) and spun for 60 s at 380 **g** to fill the card. This was checked and the card was spun again for 60 s at 380 **g** to remove any air bubbles. The card was then placed in a TaqMan Array slide sealer for sealing, and the loading ports were cut from the card before it was read in an 7900HT thermal cycler, Applied Biosystems. PCR was performed for 90 min with the following conditions: AmpErase UNG Activation for 2 min at 50°C; AmpliTaq Gold DNA Polymerase Activation for 10 min at 94.5°C; followed by 40 cycles each of Melt Anneal/Extend for 30 s at 97°C and 1 min at 59.7°C. The ‘Auto Threshold Cycle’ function was performed at the end of the run and *C*_t_ data were transferred to a Microsoft Excel spreadsheet, controls were checked, and the data were transferred to a Microsoft Access database for further analysis.

*C*_t_ values were standardised against HMBS (*PBGD*), the least variable housekeeping gene, to avoid errors due to differences in efficiency between the housekeeping and test genes, which were present from cycle 27 to 35 in most cases. A logarithmic gene expression ratio was calculated as Ln(2^−*C*t(test)^/2^−*C*t(PBGD)^) and used for comparison with ATP-TCA data by multiple linear regression using SPSS version 14.0 (SPSS Inc., Chicago, IL, USA) and Analyse-It software (Analyse-it Software Ltd, Leeds, UK) applications.

### Data analysis

The ATP-TCA and qRT-PCR data were collected into an Access database (Microsoft), from which descriptive statistics were generated. Comparison of ATP-TCA results for individual drugs or combinations with gene expression data was performed by multiple linear regression with forward selection of variables using SPSS version 15.0. For each variable, inclusion was dependant upon a probability of F>0.1, that is, the threshold for inclusion of a gene into the model within SPSS, based on initial assessment of the most appropriate model size using Akaike Information Criterion, a function of model error and size that penalises large models (data not shown). The prediction residual sum of squares method was used as this is an adjusted regression method helpful in preventing overfitting of the data as it uses a ‘leave one out’ method of analysis. Genes were added by forward regression according to their univariate correlations following entry of each gene into the model, and no intercept term was included.

## Results

### ATP-TCA

**Figure 1:**
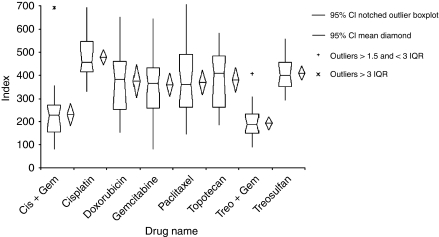
Box and whisker plot showing the sensitivity of tumours to each drug tested. Index_SUM_ is higher in resistance, lower in sensitive tumours.

In keeping with previous results from a number of articles (Andreotti *et al*, 1995; Sharma *et al*, 2003), the results from the ATP-TCA show considerable heterogeneity for each of the drugs and combinations tested. The frequency histograms show the greatest activity (i.e. lowest Index_SUM_) for treosulfan+gemcitabine, whereas the single agents showed weaker but more variable activity (Figure 1).

### qRT-PCR

Although these qRT-PCR studies were performed using RNA extracted from FFPE tissue, all samples were regarded as evaluable on the basis of the housekeeping gene *C*_t_ levels (i.e. HMBS/*PBGD C*_t_>37 cycles). Variation in housekeeping gene levels was limited, with a normal distribution of *C*_t_ levels (HMBS/*PBGD*, mean=31.61, 95% CI=30.43–32.78). *C*_t_ levels within the detectable range were present for most of the genes present on the TLDA, though some were more rarely expressed than others. NTC remained undetectable throughout the study indicating that the reagents and cards in use had not been contaminated. Control data showed an intra-assay coefficient of variation (CoV) for one sample of 0.69% for *PBGD*/HMBS. The inter-assay variation for *PBGD*/HMBS with six samples varied between 1 and 3%.

### Correlation of mechanisms with ATP-TCA data

**Figure 2:**
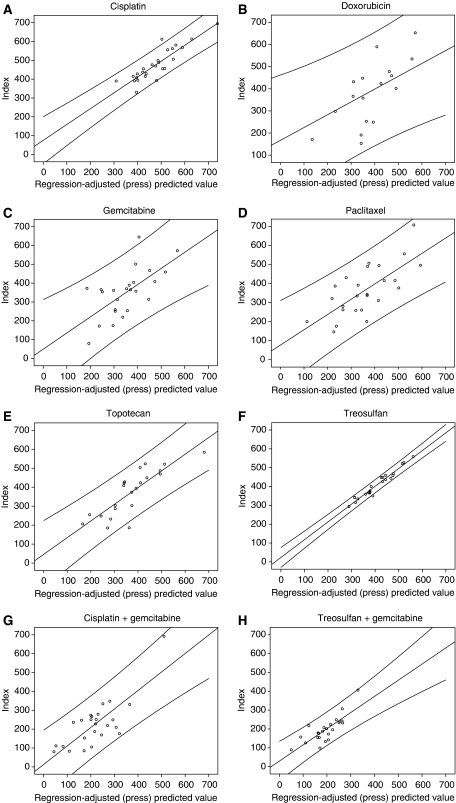
Multiple linear regression analysis by unsupervised forward selection for (**A**) cisplatin − Adj *R*^2^=0.836, *P*<0.001; (**B**) doxorubicin − Adj *R*^2^=0.524, *P*<0.007; (**C**) gemcitabine − Adj *R*^2^=0.524, *P*<0.001; (**D**) paclitaxel − Adj *R*^2^=0.646, *P*<0.001; (**E**) topotecan − Adj *R*^2^=0.751, *P*<0.001; (**F**) treosulfan − Adj *R*^2^=0.977, *P*<0.001; (**G**) cisplatin+gemcitabine − Adj *R*^2^=0.743, *P*<0.001; (**H**) treosulfan+gemcitabine − Adj *R*^2^=0.753, *P*<0.001.

Drugs susceptible to particular resistance mechanisms showed good correlation with genes linked to these mechanisms (Figure 2), provided that the drugs were sufficiently active and showed heterogeneity of chemosensitivity, though doxorubicin and paclitaxel were less highly correlated with gene expression in comparison with other drugs or combinations in multiple linear regression analyses. All of the genes on the TaqMan Arrays were previously related to drug resistance or sensitivity, and this cannot therefore be regarded as a naive data set, but for the purpose of this report the SPSS analysis included all genes present on the card. The genes involved in each of the forward multiple regression models identified and the coefficients for each are shown in Table 3 in the order of greatest contribution to the model.

Although the number of tumours is relatively small for each model, the results are impressive. The cisplatin and treosulfan models contain a number of DNA repair genes and others associated with apoptosis pathways, consistent with the known resistance mechanisms for DNA-damaging agents. The topotecan model includes a drug pump, including MRP4, and several of the models include p21 or p53. Weaker correlations were noted for doxorubicin and paclitaxel, perhaps suggesting that genes that were not represented on the array may be involved in ovarian cancer sensitivity and resistance to these drugs.

The combination of cisplatin with gemcitabine was influenced by the presence of one highly resistant tumour, and by the limited variation between the remainder of the results. However, this did not appear to be a problem for the treosulfan with gemcitabine model, which achieved a high degree of correlation despite the presence of a very resistant tumour.

## Discussion

In this study we have examined single-agent and combination effects for the same tumours using the ATP-TCA, and compared this experimental *in vitro* chemosensitivity data with the expression of a large number of potential resistance mechanisms using qRT-PCR to study FFPE biopsy material. The TaqMan Array assay can be performed with a few nanograms of RNA extracted from FFPE blocks stored in pathology, and this gives it a considerable advantage over the ATP-TCA that requires large amounts of fresh tissue. Most of the samples used in this study were from surgical material, but in each case we have used just two 1.0 mm punches from the FFPE blocks. The ability to use small punches of tumour identified by the pathologist in paraffin blocks makes this a potentially very valuable technique, as it is also be possible to measure gene amplification or mutation by PCR of DNA markers. If gene expression for sensitivity and resistance genes proves also to be related to clinical outcome, it should be feasible to develop arrays using smaller numbers of gene that can predict the efficacy of chemotherapy regimens for individual patients and to optimise patient treatment on this basis. However, it should be noted that the gene signatures identified in this study are models for acquired resistance from a very mixed poor prognosis group of patients with ovarian cancer and unlikely to be applicable to a first-line therapy setting.

Unlike direct correlation of gene expression with clinical data, the comparison of quantitative gene expression data (CoV 2%) with *in vitro* chemosensitivity data from the ATP-TCA (CoV 15%) means that relatively small numbers of tumours are required to obtain data on the genes relevant to resistance and sensitivity to drugs tested in the assay. This may prove to be particularly useful to investigate the mechanisms of sensitivity and resistance for drugs that are rarely used as single agents, and to develop predictive molecular assays for new drugs about to enter the clinical use. It is undoubtedly true that our array does not include all the genes of relevance to the drugs tested – the lack of good correlation with some agents may reflect this, or general resistance that would result in poorer correlation due to a lack of heterogeneity. However, much of the previous work on the influence of gene expression on chemosensitivity has been carried out in cell lines, which show differences in their chemosensitivity to tumour-derived cells tested in xenografts (Fiebig *et al*, 2004) or primary cultures (Fernando *et al*, 2006). Because the array was manufactured, several new correlations of gene expression with drugs active in ovarian cancer have since been described, for example gemcitabine in which ribonucleotide reductase subunits may be involved (Smid *et al*, 2006), and paclitaxel in which YY1 expression has been implicated (Matsumura *et al*, 2009). Further work is therefore required to define gene sets that might be clinically useful.

The genes identified are involved in several known mechanisms of chemoresistance, including drug metabolism, membrane drug pumps, and DNA repair. These are relatively specific to particular drugs, but in nearly all of the models generated, expression of genes involved in apoptosis proved to be important, suggesting that the general susceptibility of the cell to undergo apoptosis may outweigh other determinants of tumour chemosensitivity (Cree *et al*, 2002a; Glaysher *et al*, 2009). In general, the genes we have found to be important match well with those thought to be important from previous studies. There are some discrepancies, for instance in which membrane pumps are involved in doxorubicin or topotecan resistance. Such instances may be explained by covariance, as several such pumps tend to be up-regulated together following exposure of ovarian cancer cells to pumped drugs (Di Nicolantonio *et al*, 2005). The number of tumours in this study is relatively small, and it is important not to draw too much from these data – the likelihood of the signatures described here being optimal for prediction of chemosensitivity is remote, and much larger studies using clinical samples will be required to validate signatures for clinical use. It is however conceivable that gene signatures may be similar for patients with resistance to particular agents. Sub-clustering of groups with distinct mechanisms of resistance is conceivable in bigger studies, and could allow stratification of patients in clinical trials.

## Conclusion

This study supports the hypothesis that the molecular basis of the observed difference in sensitivity between ovarian tumours lies within the known resistance mechanisms inherent to these patients' tumours, as we have described in lung cancer (Glaysher *et al*, 2009). The TaqMan Array is well suited to investigate the presence of these mechanisms in ovarian cancers alongside cellular chemosensitivity testing and clinical results, and its ability to use small fragments of tumour tissue makes it suited to development for future clinical use.

## Figures and Tables

**Table 1:** Drug list for ATP-TCA with manufacturer and 100% TDC used in the assay and the number of tumours tested with each drug

**Drug or combination**	**Manufacturer**	**100% TDC (*μ*M)**	**Number tested**
Cisplatin	Bristol-Myers-Squibb^a^	10.00	27
Doxorubicin	Eli Lilly^b^	0.92	19
Gemcitabine	Eli Lilly	40.04	26
Paclitaxel	Bristol-Myers-Squibb	15.90	26
Topotecan	GlaxoSmithKline^c^	1.78	23
Treosulfan	Medac^d^	71.86	23
Cisplatin+Gemcitabine	As single agents	As single agents	27
Treosulfan+Gemcitabine	As single agents	As single agents	26

Abbreviation: TDC=test drug concentration.

Some samples had too few cells for all single agents and combinations to be tested.

a Bristol-Myers Squibb Pharmaceuticals Limited, Uxbridge, Middlesex, UK.

b Eli Lilly and Company Limited, Basingstoke, Hampshire, UK.

c GlaxoSmithKline plc, Brentford, Middlesex, UK.

d medac GmbH, Hamburg, Germany.

**Table 2:** Genes present on the TaqMan Array with their likely contribution to drug resistance mechanisms

**Apoptosis**	**Pumps/Detox**	**DNA repair**	**Proliferation**
*AKT*	*ATP7B*	*ATM*	*APC C-term*
*APAF1*	*BCRP*	*BRCA1*	*APC N-term*
*BAD*	*CES1*	*ERCC1*	*β-TUBULIN III*
*BAX*	*CES2*	*ERCC2*	*COX2*
*BCL2*	*cN II*	*GTF2H2*	*EGFR*
*BCL-x(L)*	*DPD*	*MGMT*	*HER2*
*BID*	*FPGS*	*MLH1*	*HER3*
*c-FLIP*	*γH2AX*	*MSH2*	*HER4*
*FAS*	*GCLC*	*MSH6*	*HIF1A*
*FASL*	*GCLM*	*RAD51*	*KI67*
*HSP60*	*GSTπ*	*TOPO I*	*P16*
*HSP70*	*hENT1*	*TOPO IIa*	*P21*
*HSP90*	*hENT2*	*TOPO IIb*	*P27*
*IAP2*	*MDR1*	*XPA*	*P53*
*IGF1*	*MRP1*	*XRCC1*	*VEGF*
*IGF1R*	*MRP2*	*XRCC5*	
*IGF2*	*MRP3*	*XRCC6*	
*IGF2R*	*MRP4*		
*IGFBP1*	*MRP5*		
*IGFBP2*	*MRP6*		
*MCJ*	*MRP8*		
*MCL1*	*MTII*		
*mTOR*	*MVP*		
*NFκB*	*OPRT*		
*PIK3CA*	*RRM1*	Housekeeping genes	
*PTEN*	*SOD1*	*18S*	
*STAT3*	*TAP1*	*HPRT*	
*SURVIVIN*	*TAP2*	*PBGD*	
*XIAP*	*TAP4*	*SDHA*	
	*TS*	*TBP*	

**Table 3:** Genes found to correlate in multivariate linear regression analysis

**Model**	***R***	***R* ^2^**	**Adj *R*^2^**	**Std. error**	**Significance (ANOVA)**	
**(A)**						
Cisplatin	0.949	0.901	0.836	35.226	*P*<0.001	
Doxorubicin	0.797	0.636	0.524	98.669	*P*<0.007	
Gemcitabine	0.766	0.586	0.524	88.312	*P*<0.001	
Paclitaxel	0.848	0.720	0.646	78.669	*P*<0.001	
Topotecan	0.900	0.811	0.751	60.457	*P*<0.001	
Treosulfan	0.994	0.989	0.977	10.769	*P*<0.001	
Cisplatin+Gemcitabine	0.899	0.807	0.743	63.769	*P*<0.001	
Treosulfan+Gemcitabine	0.904	0.817	0.753	33.823	*P*<0.001	
						
	**Unstandardized**				**Collinearity statistics**	
**Gene**	***B***	**Std. Error**	**Standardized** ***β***	***t*** **Lower bound**	***B***	**Std. error**
**(B)**						
*Cisplatin*						
(Constant)	489.826	22.739		21.541		
*Rad51*	−41.140	6.003	−0.801	−6.853	0.482	2.077
*IGF1R*	−61.361	11.034	−0.737	−5.561	0.375	2.668
*p53*	19.690	8.267	0.217	2.382	0.792	1.263
*Topo I*	43.796	9.300	0.522	4.709	0.534	1.872
*MRP5*	17.255	5.629	0.345	3.065	0.518	1.929
*Survivin*	−11.745	4.880	−0.286	−2.407	0.466	2.145
*HSP60*	42.165	9.610	0.491	4.388	0.526	1.901
*BCRP*	−21.628	5.093	−0.632	−4.247	0.297	3.368
*Mcl-1*	16.918	6.486	0.252	2.609	0.707	1.414
Ki67	11.667	5.487	0.259	2.126	0.442	2.264
						
*Doxorubicin*						
(Constant)	69.454	125.422		0.554		
*GST pi*	106.938	31.844	0.649	3.358	0.751	1.332
*MRP4*	80.498	25.470	0.585	3.161	0.817	1.225
*MRP5*	−36.958	13.921	−0.500	−2.655	0.790	1.266
*Topo IIa*	−14.009	16.405	−0.164	−0.854	0.759	1.318
						
*Gemcitabine*						
(Constant)	407.883	28.640		14.242		
(Constant)	454.312	37.512		12.111		
*MCJ*	−46.173	10.778	−0.621	−4.284	0.983	1.018
*XRCC1*	52.043	14.788	0.571	3.519	0.785	1.274
*XPA*	−48.982	19.639	−0.402	−2.494	0.797	1.255
						
*Paclitaxel*						
(Constant)	343.514	37.244		9.223		
*Survivin*	−42.179	8.466	−0.675	−4.982	0.804	1.245
*IGF2R*	109.226	18.578	1.122	5.879	0.405	2.472
*Mcl-1*	−70.805	17.654	−0.799	−4.011	0.372	2.689
*GCLM*	40.381	12.968	0.490	3.114	0.596	1.677
*TAP2*	−36.754	15.467	−0.333	−2.376	0.750	1.333
						
*Topotecan*						
(Constant)	356.986	30.510		11.701		
*APAF1*	75.074	11.458	0.805	6.552	0.785	1.274
*SDHA*	−42.701	9.085	−0.578	−4.700	0.783	1.277
*HER2*	−41.223	8.955	−0.618	−4.604	0.657	1.521
*MRP4*	55.124	15.928	0.510	3.461	0.546	1.831
*IGF2R*	36.298	15.445	0.355	2.350	0.520	1.924
						
*Treosulfan*						
(Constant)	−46.200	22.925		−2.015		
*MLH1*	42.868	1.922	0.893	22.304	0.646	1.549
*Bax*	75.608	5.919	0.927	12.774	0.197	5.083
*Bcl2*	−31.437	1.650	−1.244	−19.054	0.243	4.115
*p21*	−69.282	3.493	−1.218	−19.837	0.275	3.641
*GST pi*	106.821	6.921	1.176	15.433	0.178	5.608
*Ki67*	−17.337	1.649	−0.509	−10.511	0.441	2.266
*APC* N-term	56.943	5.738	0.870	9.924	0.135	7.417
*HIF1A*	−45.059	6.890	−0.566	−6.539	0.138	7.224
*p16*	7.875	1.539	0.208	5.117	0.629	1.589
*Survivin*	−7.258	1.554	−0.223	−4.671	0.455	2.198
*Bid*	−15.379	4.423	−0.204	−3.477	0.301	3.327
						
*Cisplatin*+*Gemcitabine*						
(Constant)	−195.787	106.134		−1.845		
*FPGS*	58.087	11.147	0.630	5.211	0.733	1.364
*IGFBP2*	−27.128	11.555	−0.275	−2.348	0.778	1.285
*MDR1*	−52.072	8.573	−0.871	−6.074	0.520	1.922
*18s*	48.716	11.831	0.578	4.118	0.543	1.841
*XRCC5*	144.209	32.907	0.859	4.382	0.278	3.591
*HIF1A*	−76.886	25.948	−0.555	−2.963	0.305	3.281
						
*Treosulfan*+*gemcitabine*						
(Constant)	280.890	22.522		12.472		
*ATP7B*	−25.392	4.143	−0.721	−6.128	0.776	1.289
*Bad*	27.786	8.089	0.401	3.435	0.787	1.271
*MTII*	−55.067	10.870	−0.648	−5.066	0.657	1.521
*p53*	34.839	8.973	0.485	3.883	0.690	1.449
*IGF2*	−12.457	3.713	−0.391	−3.355	0.791	1.265
*GCLM*	11.706	4.972	0.272	2.354	0.807	1.239

Abbreviation: ANOVA=analysis of variance.

(**A**) With ATP-TCA data for recurrent ovarian cancer: model summaries; (**B**) using forward selection: coefficients for each gene included in the model by drug or combination tested.
